# Inhibition of mTOR delayed but could not prevent experimental collapsing focal segmental glomerulosclerosis

**DOI:** 10.1038/s41598-020-65352-y

**Published:** 2020-05-22

**Authors:** Laura Miesen, Jennifer Eymael, Shagun Sharma, Markus A. Loeven, Brigith Willemsen, Marinka Bakker-van Bebber, Fieke Mooren, Catherine Meyer-Schwesinger, Henry Dijkman, Jack F. M. Wetzels, Jitske Jansen, Johan van der Vlag, Bart Smeets

**Affiliations:** 1 Department of pathology, Radboud Institute for Molecular Life Sciences, Radboud university medical center, Nijmegen, The Netherlands; 2 School of Biomedical Sciences, University of Plymouth, Plymouth, UK; 3 Department of nephrology, Radboud Institute for Molecular Life Sciences, Radboud Institute for Health Sciences, Radboud university medical center, Nijmegen, The Netherlands; 4 Institute of Cellular and Integrative Physiology, Center for Experimental Medicine, University Medical Center Hamburg -Eppendorf, Hamburg, Germany; 5 Department of pediatric nephrology, Radboud Institute for Molecular Life Sciences, Radboud university medical center, Amalia Children’s Hospital, Nijmegen, The Netherlands

**Keywords:** Drug therapy, Focal segmental glomerulosclerosis

## Abstract

Anti-Thy1.1 transgenic mice develop glomerular lesions that mimic collapsing focal segmental glomerulosclerosis (FSGS) in humans with collapse of the glomerular tuft and marked hyperplasia of the parietal epithelial cells (PECs). Immunostaining of phosphor-S6 ribosomal protein (pS6RP) revealed high mTOR activity in PECs of the FSGS lesions of these mice. In this study we questioned whether the mTOR inhibitor rapamycin (sirolimus) could attenuate the development and progression of glomerulosclerotic lesions in the anti-Thy1.1 transgenic mice. We observed reduced mTOR signalling and proliferation in human parietal epithelial cells after rapamycin treatment. Experiments with anti-Thy1.1. mice showed that early treatment with sirolimus reduced the development of glomerular lesions and glomerular cell proliferation at day 4. Levels of albuminuria, podocyte injury and podocyte number were similar in the sirolimus and vehicle treated groups. The initial beneficial effects of sirolimus treatment were not observed at day 7. Late sirolimus treatment did not reduce albuminuria or the progression of glomerulosclerosis. Taken together, rapamycin attenuated PEC proliferation and the formation of early FSGS lesions in experimental FSGS and reduced human PEC proliferation *in vitro*. However, the initial inhibition of PEC proliferation did not translate into a decline of albuminuria nor in a sustained reduction in sclerotic lesions.

## Introduction

Focal segmental glomerulosclerosis (FSGS) is characterized by the formation of sclerotic lesions in the glomeruli of the kidneys. FSGS is one of the most common glomerular disorders and the leading cause of end-stage renal disease (ESRD) in the United States^1^. Several underlying conditions can lead to FSGS such as diabetes, hypertension and obesity. In addition, FSGS can be caused by genetic mutations affecting the function of essential glomerular cell proteins or it can be idiopathic^1,2^. The diagnosis of FSGS is largely based on histopathological findings characterized by the adhesions of the Bowman’s capsule with the glomerular tuft, the formation of focal and segmental sclerotic lesions, obliteration of glomerular capillaries and extracellular matrix accumulation^3,4^. Currently, the different histological patterns of FSGS have been divided into five subvariants: the perihilar-, the tip -, the cellular-, the NOS (not otherwise specified)-, and the collapsing variant, latter is characterized by collapse of the glomerular tuft and PEC hyperplasia^2^.

The pathogenesis of FSGS is not completely unravelled. In the last decade we and others demonstrated that parietal epithelial cells (PECs) are crucially involved in the formation of sclerotic lesions. Genetic tagging of podocytes or PECs in different models of FSGS showed that upon induction of glomerular injury PECs migrate to the capillary tuft and induce the adhesion between Bowman’s capsule and the tuft^5,6^. These activated PECs invade the injured area and deposit extracellular matrix that results in the progression of FSGS^7,8^. In addition, similar pathohistological patterns were observed in secondary FSGS that are independent of the underlying disease^9^. These findings strengthen the assumption that common molecular pathways lead to PEC activation and subsequently to FSGS formation and progression.

Hamatani and colleagues observed activation of the mammalian target of rapamycin (mTOR), a main player in cell proliferation and survival, specifically in PECs in experimental models of glomerular diseases. They observed that a reduction of the stress-inducible protein sestrin-2 in PECs was associated with increased pS6RP expression as well as de *novo* expression of the activation marker CD44^10^. The results from Hamatani *et al*. suggested that mTOR signalling plays an important role in the switch from resting to activated PECs seen in progressive glomerular disease. Therefore, mTOR might be a pharmaceutical target to prevent or reduce PEC activation and subsequently glomerulosclerosis formation.

In the present study we investigated this hypothesis and studied the effects of mTOR inhibition on immortalized human parietal epithelial cells and in anti-Thy1.1 transgenic mice, a model resembling collapsing FSGS in humans.

## Results

### Rapamycin reduces mTOR signalling and cell proliferation of human parietal epithelial cells

**Figure 1:**
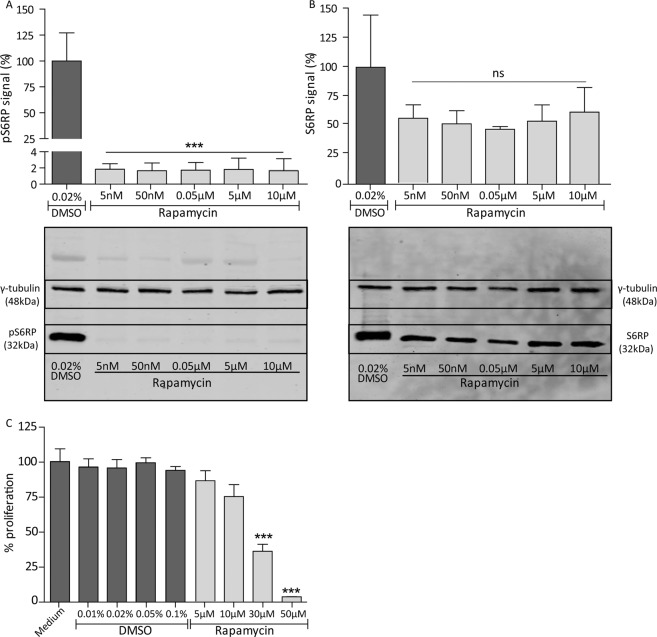
Rapamycin inhibits mTOR signalling and cell proliferation of human immortalized PECs. (**A**) mTOR signalling is presented as pS6RP expression. Protein expression of pS6RP was significantly reduced in PECs treated with 5nM-10µM rapamycin compared to cells treated with 0.02% (v/v) DMSO (vehicle) for 24 hours. Quantitative analysis was performed using the results of blots of 3 different experiments (n = 3). An example of the protein expression of γ-tubulin and pS6RP of one of the three western blots is shown underneath the graph. Protein expression is shown from cells treated with the DMSO control and 5 nM–10 µM rapamycin. The signal of γ-tubulin and pS6RP shown are cropped and marked with a black box. All full-length blots are presented in Supplementary Fig. S5. (**B**) No significant reduction of the non-phosphorylated S6RP was observed in rapamycin treated cells after 24 hours. Quantitative analysis was performed using the results of blots of 3 different experiments (n = 3). An example of the protein expression of γ-tubulin and S6RP of one of the three western blots is shown underneath the graph. Protein expression is shown from cells treated with the DMSO control and 5 nM-10 µM rapamycin. The signal of γ-tubulin and S6RP shown are cropped and marked with a black box. All full-length blots are presented in Supplementary Fig. S6. (**C**) Cell proliferation was significantly inhibited by rapamycin (30 µM, 50 µM, 24 hours) compared to controls. Mean with SEM is shown (n = 3). ***P ≤ 0.001, ns P ≥ 0.05.

Since we hypothesized that mTOR signalling is important for PEC proliferation in FSGS, we investigated the effect of the mTOR inhibitor rapamycin on cultured human immortalized PECs. The expression of pS6RP as readout for mTOR signalling and PEC proliferation was studied, when exposed to different concentrations of rapamycin or DMSO as control. Already at a concentration of 5 nM rapamycin the expression of the pS6RP was reduced (Fig. 1A, p < 0.001). The non-phosphorylated protein was not significantly affected (Fig. 1B). In addition, higher concentrations of rapamycin (≥30 µM) reduced PEC proliferation (Fig. 1C, p < 0.001). These findings show that rapamycin can target PECs directly *in vitro*. As PEC proliferation is described to be related to CD44 expression^11^, the effect of rapamycin on CD44 gene and protein expression was studied (Supplementary Fig. S1). The results indicate that the inhibition of PEC proliferation after rapamycin treatment is independent of the expression level of CD44.

### mTOR activity is increased in sclerotic lesions

To study the effect of mTOR inhibition in PECs *in vivo* we used the transgenic anti-Thy1.1 mouse, an experimental model for collapsing FSGS. Injection of the anti-Thy1.1 antibody in the transgenic Thy1.1 mice results in an almost immediate development of albuminuria that peaks at day 1 after injection^12^. Within 7 days the FSGS lesions, that resemble collapsing FSGS and are associated with hyperplasia of the PECs^5,12,13^, are formed. Early stages of the lesions can already be observed after 4 days^13^.

**Figure 2:**
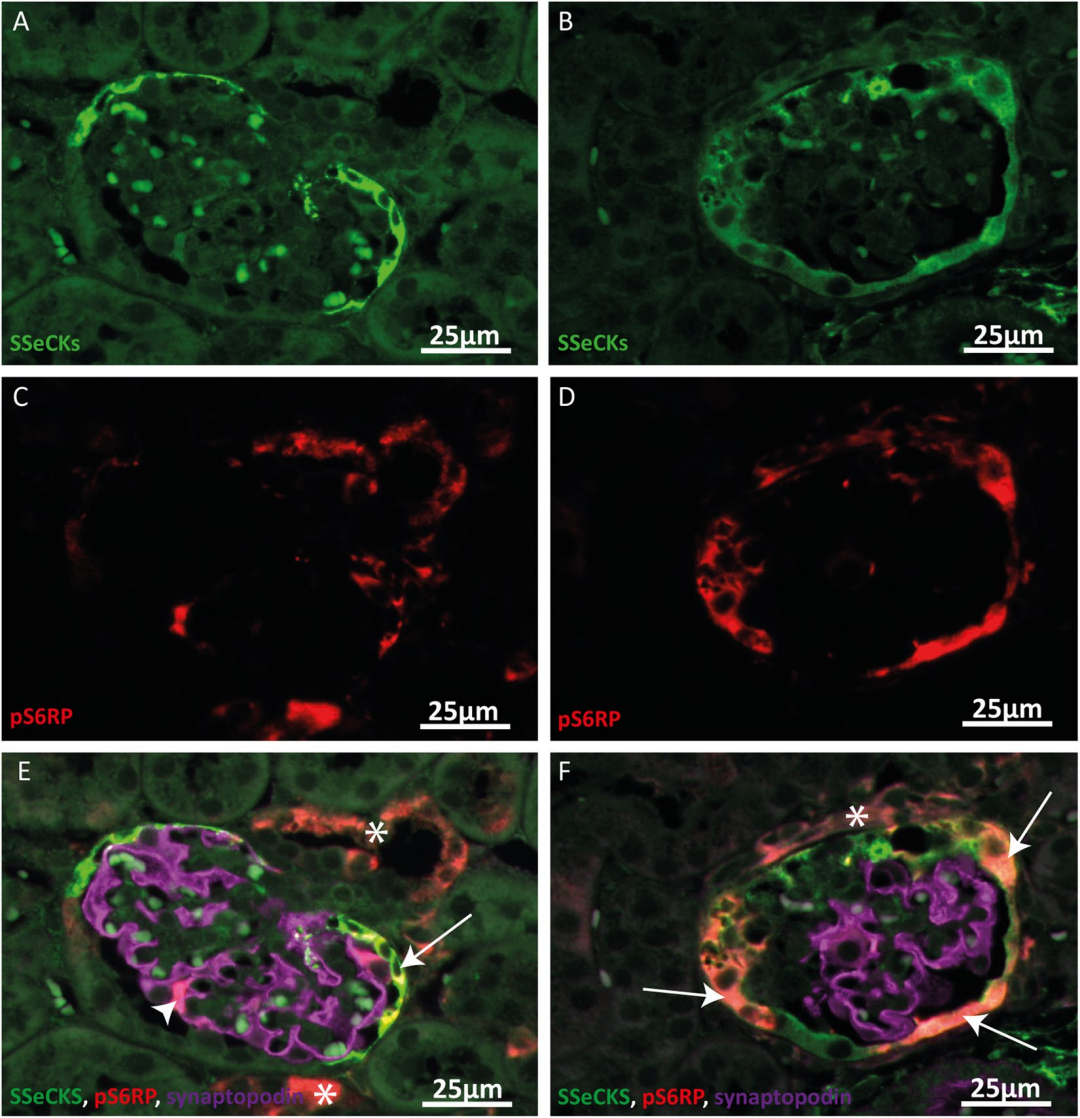
Increased pS6RP expression in parietal epithelial cells in sclerotic glomeruli. (**A, C, E**) Representative images of a morphological normal glomerulus of an anti-Thy1.1 mouse 4 days after disease induction. SSeCKS (green, A, E), pS6RP (red, C, E) and synaptopodin (violet, E) expression is shown. pS6RP expression was observed in some SSeCKS positive cells (arrow) and in some synaptopodin positive podocytes (arrowhead) and outside of the glomerulus (asterisk). (**B, D, F**) Representative images of a sclerotic glomerulus of an anti- Thy1.1 mouse 4 days after disease induction. In sclerotic glomeruli an increased pS6RP expression in SSeCKS positive PECs was observed (arrows). pS6RP expression could also be detected outside of the glomerulus (asterisk).

Immunostaining of the podocyte marker synaptopodin, the PEC marker SSeCKS^14^ and pS6RP as marker for mTOR signalling revealed an increased pS6RP expression in PECs in the affected glomeruli of the anti-Thy1.1 mice, while in morphologically normal glomeruli only few cells showed mTOR signalling (Fig. 2). These results suggest that increased mTOR activity may play a role in PEC activation and disease progression in the anti-Thy1.1. mice.

### Sirolimus treatment does not reduce albuminuria and podocyte damage

**Figure 3:**
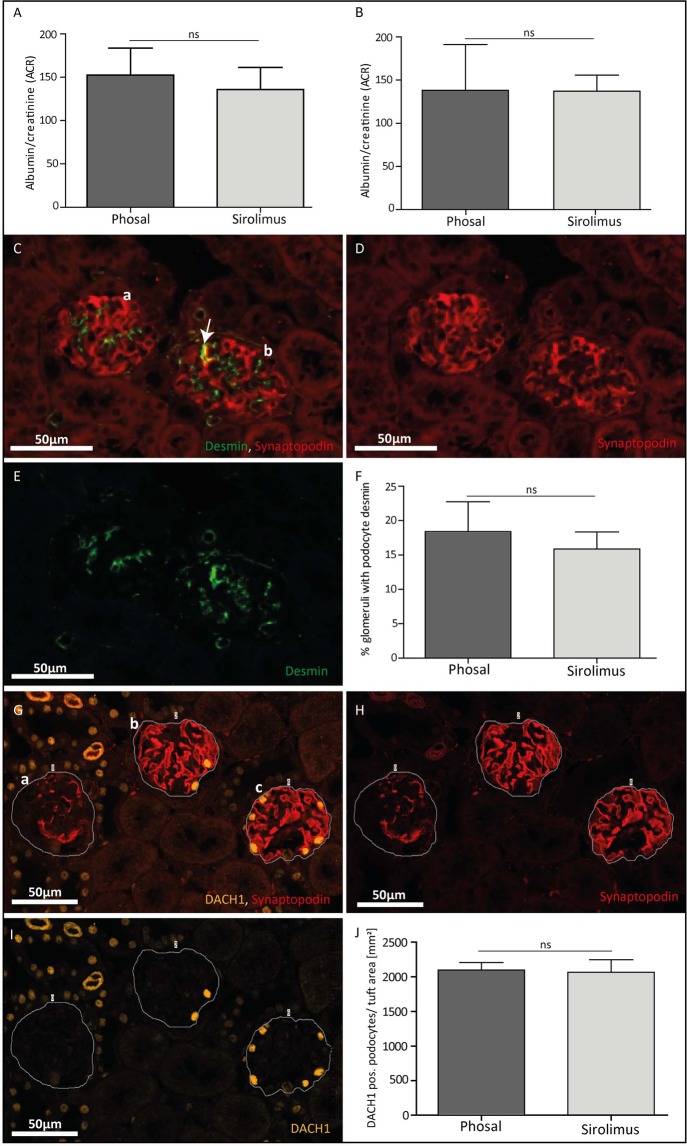
Sirolimus treatment of experimental FSGS did not change albuminuria, podocyte injury and podocyte number. (**A**) Sirolimus treated mice did not show a lower albumin/creatinine ratio compared to control mice at day 4 and (**B**) day 7. Sirolimus treated mice n = 12, phosal treated mice n = 8. Mean with SD is shown. ns P ≥ 0.05. (**C, D, E**) Representative images of glomeruli stained against synaptopodin (C, D, red) and desmin (C, E, green). (**C**) A healthy glomerulus (a) only shows desmin expression in the mesangial cells. In diseased glomeruli (b) desmin staining can also be detected in podocytes (co-localizing with synaptopodin expression (C, yellow, arrow)). (**F**) Glomerular desmin expression was similar in the sirolimus and control group. Sirolimus treated mice n = 4, phosal treated mice n = 4. Mean with SD is shown. ns P ≥ 0.05. (**G, H, I**) Representative images of glomeruli stained against synaptopodin (G, H, red) and DACH1 (G, I, yellow). (**G**) A line was drawn surrounding the tuft area to measure it. The whole tuft area was selected also in case no or less synaptopodin signal was present (example glomerulus a). (**J**) Phosal and sirolimus treated mice have a similar number of DACH1 positive podocytes per mm^2^ of the tuft area. Sirolimus treated mice n = 6, phosal treated mice n = 6. Mean with SEM is shown. ns P ≥ 0.05.

We evaluated in the anti-Thy1.1 mouse model the effect of sirolimus on the development of the first visible glomerular lesions at day 4 and the fully developed lesions at day 7. Following sirolimus treatment, albuminuria was not reduced at day 4 (Fig. 3A) and day 7 (Fig. 3B), while binding of the anti-Thy1.1 antibody was equal in the sirolimus and phosal (vehicle) treated mice (data not shown). Immunofluorescent staining for synaptopodin and the podocyte injury marker desmin (Fig. 3C–E) showed no differences in the amount of podocyte injury between the phosal and sirolimus treated mice at day 4 (Fig. 3F). In addition, immunofluorescent staining for DACH1 in combination with synaptopodin (Fig. 3G–I) revealed that the number of podocytes was similar in sirolimus and phosal treated mice (Fig. 3J). These results indicate that early mTOR inhibition did not have an effect on the degree of podocyte injury, podocyte number and proteinuria in the anti-Thy1.1 mouse model.

### Sirolimus reduces glomerular damage and cell proliferation at day 4

**Figure 4:**
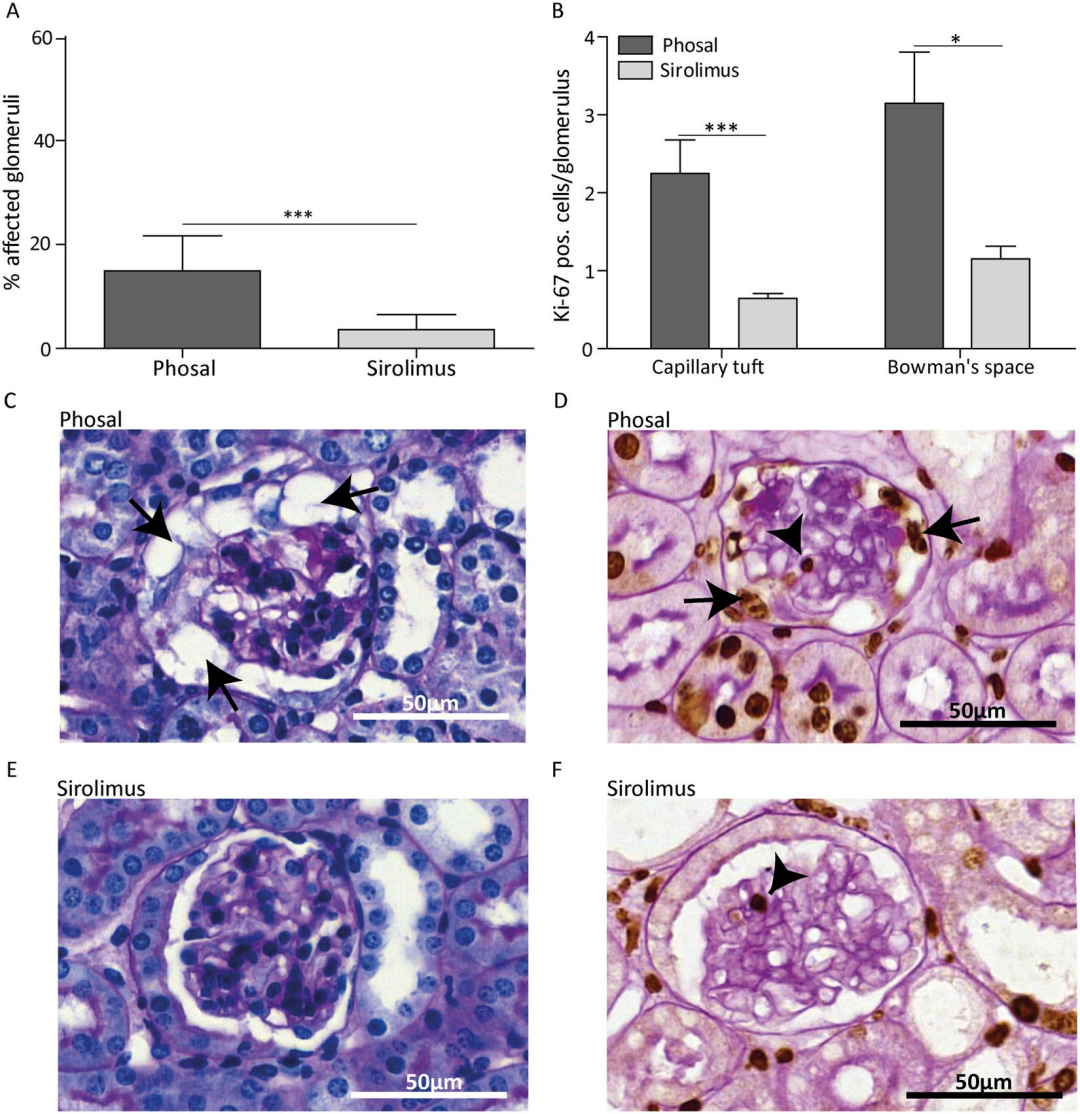
Sirolimus treatment reduced glomerular damage and cell proliferation at day 4 in experimental FSGS. (**A**) Sirolimus treated mice showed significant less affected glomeruli compared to phosal treated mice. At least 55 glomeruli per mouse were scored. Sirolimus treated mice n = 11, phosal treated mice n = 10. (**B**) Proliferation is expressed as ki-67 positive cells per glomerulus. Ki-67 positive cells were counted in at least 55 glomeruli per mouse. Cell proliferation in the tuft and in the Bowman’s space was significantly reduced at day 4. Sirolimus treated mice n = 12, phosal treated mice n = 10. (**C, E**) Representative images of glomeruli stained with PAS of phosal and sirolimus treated mice. Arrows indicate vacuolization in the glomerulus. (**D, F**) Representative images of glomeruli stained with ki-67 (brown colour) and PAS without hematoxylin stain. Arrows indicate cell proliferation in the Bowman’s space, arrowheads indicate cell proliferation in the glomerular tuft. Mean with SEM is shown. *P ≤ 0.05, ***P ≤ 0.001.

Despite similar levels of albuminuria and podocyte injury between the phosal and sirolimus treated mice, we observed less affected glomeruli in mice treated with sirolimus compared to the control mice at day 4 (Fig. 4A). Glomeruli were considered affected when they showed one of the following histological changes: vacuolization of the epithelial cells, hyalinosis or epithelial cell hyperplasia (Supplementary Fig. S2). The majority of phosal treated mice had a more severe histological phenotype showing more protein casts and affected glomeruli (Fig. 4C). The affected glomeruli of sirolimus treated mice mainly appeared with vacuolization and mild cell proliferation. However, the greatest part of glomeruli was unaffected (Fig. 4E). In addition, we detected fewer proliferating (ki-67 positive) cells in the Bowman’s space in sirolimus treated mice, indicating less PEC proliferation (Fig. 4B, D, F). The sirolimus treated mice also showed fewer proliferating cells in the glomerular tuft (Fig. 4B). As proliferating endothelial cells were identified in the mice (Supplementary Fig. S3), their proliferation was probably also reduced due to sirolimus treatment. *In vitro* we showed that rapamycin indeed has a direct effect on the glomerular endothelial cell proliferation (Supplementary Fig. S4).

### Sirolimus does not reduce glomerular damage and cell proliferation at day 7

**Figure 5:**
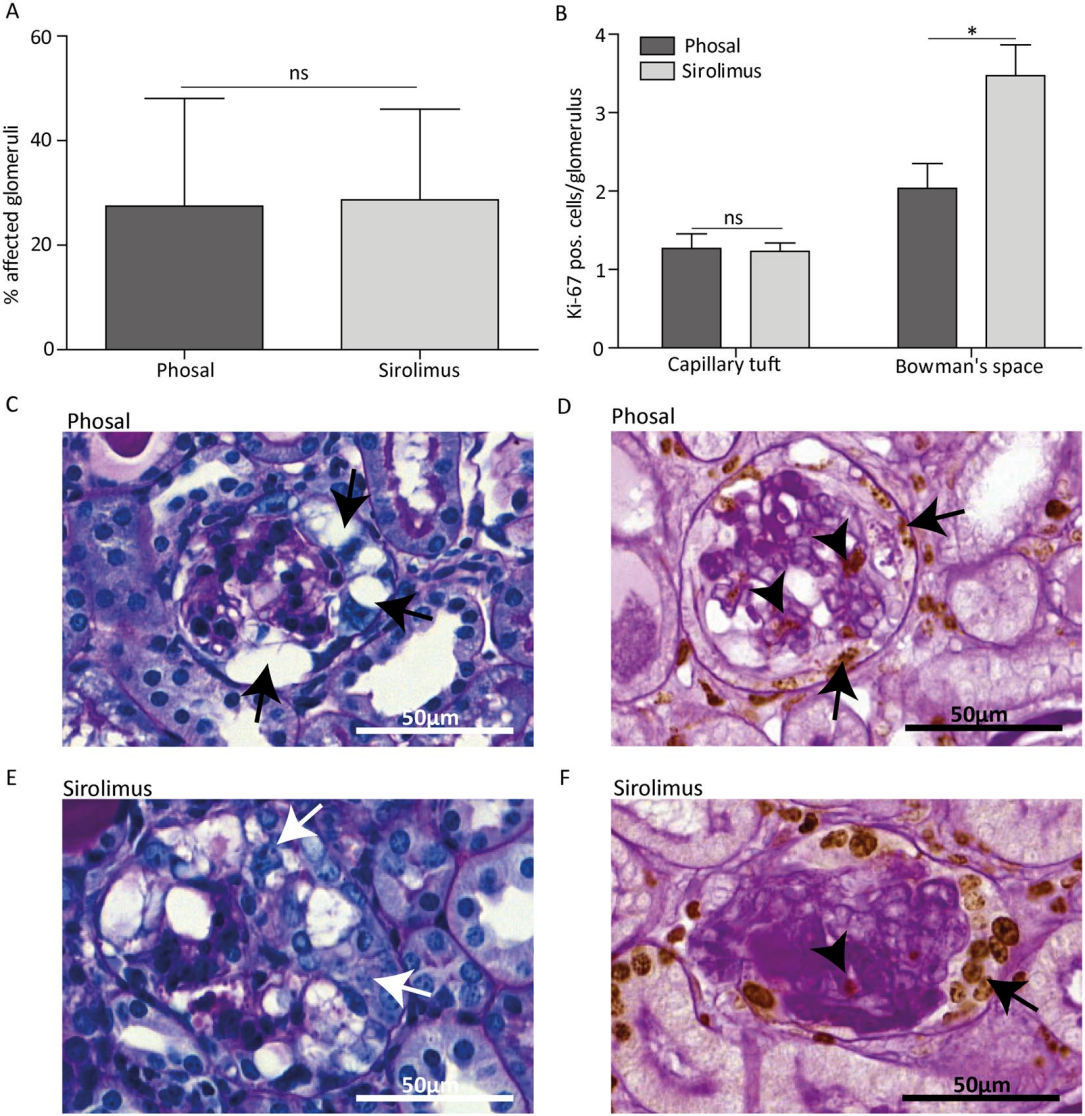
Sirolimus does not reduce cell proliferation and sclerosis at day 7 in experimental FSGS. (**A**) Sirolimus treated anti-Thy1.1 mice showed no significant differences in the percentage of affected glomeruli compared to phosal treated mice. At least 55 glomeruli per mice were scored. Sirolimus treated mice n = 13, phosal treated mice n = 9. (**B**) Proliferation is expressed as ki-67 positive cells per glomerulus. Ki-67 positive cells were counted in at least 55 glomeruli per mouse. Sirolimus treated mice showed similar cell proliferation in the tuft and more cell proliferation in the Bowman’s space compared to phosal treated mice. Sirolimus treated mice n = 13, phosal treated mice n = 9. (**C, E**) Representative images of glomeruli stained with PAS of phosal and sirolimus treated mice. Black arrows indicate vacuolization of the glomerulus. White arrows indicate PEC proliferation. Hyalinosis deposits can be observed in the capillary tuft (dark violet). (**D, F**) Representative images of glomeruli stained with ki-67 (brown colour) and PAS without hematoxylin stain. Arrows indicate cell proliferation in the Bowman’s space, arrowhead indicates cell proliferation in the glomerular tuft. Dark violet colour in the capillary tuft indicates hyalinosis. Mean with SEM shown. *P ≤ 0.05, ns P ≥ 0.05.

As sirolimus reduced PEC proliferation and the formation of lesions during the early proliferative phases of FSGS development, we next investigated whether early sirolimus treatment sustained reduction of sclerotic lesions. However, the number of affected glomeruli at day 7 in the phosal and sirolimus treated groups was similar (Fig. 5A). Histological analysis revealed that hyalinosis was clearly visible in the glomeruli and that tubular protein casts could be observed in both the phosal and sirolimus treated mice. In addition, the majority of affected glomeruli showed extensive PEC proliferation (Fig. 5C–F). Notably, the sirolimus treated mice showed the highest number of ki-67 positive cells in Bowman’s space (Fig. 5B).

### Sirolimus treatment after glomerulosclerosis development does not reduce albuminuria and sclerosis

**Figure 6:**
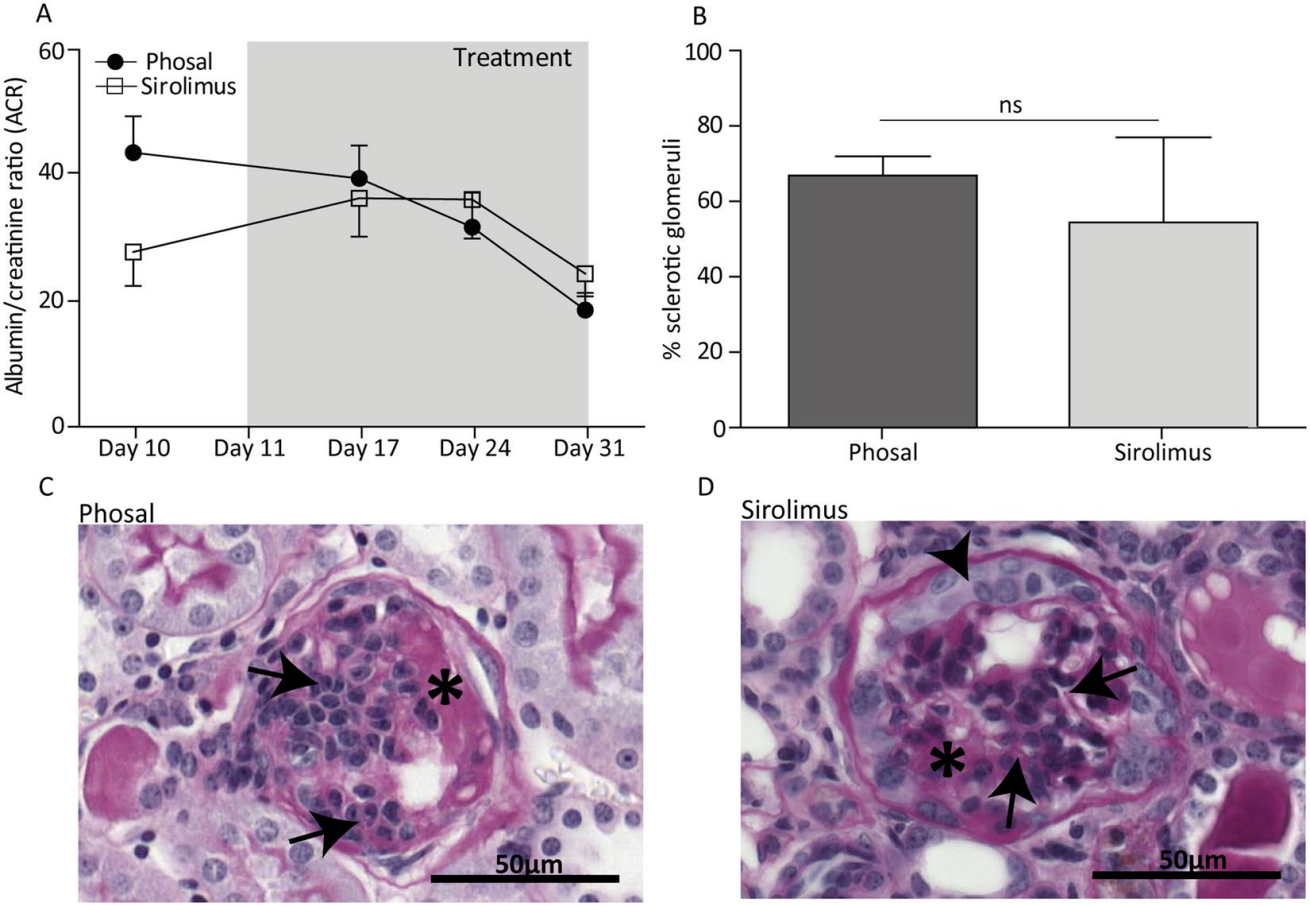
Late sirolimus treatment did not attenuate albuminuria and glomerulosclerosis in anti-Thy1.1 mice at day 31. (**A**) Albumin/creatinine ratio of phosal and sirolimus injected mice. No significant differences between the mice could be measured over time. Sirolimus treated mice n = 14, phosal treated mice n = 15. (**B**) The number of sclerotic glomeruli was not significant different between the sirolimus treated and control mice (day 31). At least 71 glomeruli per mouse were scored. Sirolimus treated mice n = 14, phosal treated mice n = 11. (**C**, **D**) Representative images of glomeruli stained with PAS of a (**C**) phosal treated mouse and a (**D**) sirolimus treated mouse. The histology between the phosal and sirolimus treated mice was comparable showing glomerulosclerosis (arrows), hyalinosis (asterisks) and PEC hyperplasia (arrowhead). Mean with SEM shown. ns P ≥ 0.05.

To evaluate whether mTOR inhibition can attenuate the progression of glomerulosclerosis in a later phase of disease development, we performed an experiment treating anti-Thy1.1 mice daily with sirolimus or phosal for a period of 20 days. Knowing that sclerotic lesions are formed within the first 7 days after anti-Thy1.1 antibody injection^13^, we started sirolimus and phosal treatment at day 11. Comparing the albuminuria levels of the sirolimus and phosal treated mice, no significant differences could be observed (Fig. 6A). Furthermore, the number of sclerotic glomeruli was similar (Fig. 6B). In general, the kidney tissue of all mice showed influx of immune cells in the glomeruli and the interstitium. Hyalinosis and collapsing tufts could be detected in the glomeruli (Fig. 6C, D). Some glomeruli still displayed some PEC proliferation. The results indicate that mTOR inhibition did not have an effect on the glomerular pathology once the glomerular lesions are formed. In general, our findings indicate that sirolimus reduces PEC proliferation and formation of lesions in the early proliferative phase of FSGS development but cannot prevent the further development of glomerulosclerosis.

## Discussion

In this study, we tested the hypothesis that mTOR signalling regulates PEC activation and that mTOR inhibition may prevent PEC proliferation and subsequently glomerulosclerosis in the anti-Thy1.1 mouse model, an experimental model of collapsing FSGS.

We observed an increased mTOR activity, expressed as the phosphorylation of S6RP, in affected glomeruli of the anti-Thy1.1 mice. Especially PECs showed a striking increase in pS6RP expression. Sirolimus treatment reduced the number of affected glomeruli, which was associated with less PEC proliferation, in the early lesions seen at day 4. The reduced PEC proliferation could be a direct effect of rapamycin on PECs, since also rapamycin treated PECs in culture showed reduced pS6RP levels and reduced proliferation. In addition, the effects on PECs were independent of podocytes as the sirolimus treatment did not have an effect on albuminuria, the number of podocytes or podocyte injury. However, despite the marked effects of rapamycin in culture and in the development of the early lesions in the mice, continuation of the treatment with sirolimus could not prevent glomerulosclerosis at day 7. Also sirolimus treatment after sclerosis development could not reduce lesion formation.

Recent studies indicated that in the formation of lesions in FSGS and crescentic glomerulonephritis the PECs become activated^5,9^. The normal quiescent flat cells become enlarged and show different marker expression, i.e. a *de novo* expression of CD44^8,11,15^. Although, we and others have established the involvement of PECs in the development of glomerular lesions, still little is known about the molecular processes driving PEC activation^16^. Several studies have described a role for mTOR in glomerular disease models. Although, most of these studies focused on mTOR signalling in podocytes^17–21^, a few studies have studied the pathway in PECs^10,22,23^. These studies indicated that mTOR signalling might be involved in PEC activation and therefore the development of sclerotic lesions.

This study shows a link between mTOR signalling and PEC activation as well. Inhibiting mTOR in the anti-Thy1.1 mice reduced PEC proliferation and sclerosis formation at day 4. However, it was striking that the beneficial effects of mTOR signalling were not seen in later phases of the disease. These contradictory findings can also be seen in other studies that investigated mTOR signalling in PECs. In general, all studies, including this work, showed that the effects of mTOR inhibition are dependent on the dose of the mTOR inhibitor, the animal model used, the route of administration and on the onset and duration of treatment.

Similar to our observations, Kurayama *et al*. showed that the outcome of mTOR inhibition is dependent on the onset and duration of the mTOR inhibition. The study demonstrated that in a rat model of crescentic nephritis early treatment (day of disease induction) with everolimus led to increased cellular necrosis. However, later treatment (7 days after disease induction) reduced glomerular crescent formation^22^. Since the anti-Thy1.1 model and the nephritis model are different models of treatment strategies and time, the observed effects are difficult to compare to our findings. Nevertheless, both studies show that the timing of treatment is critical.

Besides the timing, also the dose of rapamycin is of importance for the treatment results. From seminal studies performed by Gödel and co-workers it has become clear that mTOR signalling is tightly regulated and that an imbalance in mTOR activity may lead to adverse effects, facilitating glomerular disease^17^. In their study Gödel and colleagues deleted the regulatory-associated protein (Raptor), an essential adaptor molecule of mTOR, in podocytes of diabetic mice. They demonstrated that complete reduction of mTORC1 in Raptor^Δpodocyte^ mice resulted in proteinuria and the progression of diabetic nephropathy, while deletion of only one allele of Raptor (Raptor^Het podocyte^) inhibited mTOR activation, leading to decreased proteinuria and glomerulosclerosis^17^. Hence, it was demonstrated that complete reduction of mTOR activity can be responsible for the progression of glomerular injury. This means that the reduction of mTOR activity to prevent disease progression should be done with care, as a too strong reduction can have deleterious effects. This conclusion can also be drawn from a similar study performed by Zschiedrich *et al*. They showed that a homozygous podocyte specific knockout (KO) of Raptor in an adriamycin induced nephropathy mouse model caused massive proteinuria and sclerosis, while a heterozygous KO resulted in a reduction of proteinuria and sclerosis^21^. They also showed that a high dose of administered rapamycin has a similar effect as a complete Raptor KO, while low rapamycin concentrations resembled the beneficial outcome of the heterozygous KO, again stressing the tight regulation of mTOR in the glomerulus.

The possible decrease in endothelial proliferation due to mTOR inhibition might have contributed to the recurrent sclerosis formation observed in our experiment, as reduced endothelial cell proliferation presumably impairs endothelial repair of capillaries that normally is required after glomerular damage due to anti-Thy1.1 injection^24^.

Due to the narrow therapeutic window of sirolimus, and in the murine studies described problems of treatment onset and duration, the implementation of sirolimus into the clinic to treat glomerulosclerosis will be challenging. Sirolimus and other rapamycin analogues (rapalogous) are approved by the Food and Drug administration (FDA) for the treatment of cancers, transplant rejection, lymphangioleiomyomatosis and tuberous sclerosis complex^25^. Therapeutic effects of sirolimus or rapalogous are often hampered due to recurrence of the disease assumably caused by negative feedback loops. To prevent this, treatments combining sirolimus with drugs targeting the pathways involved in the negative feedback (e.g. Akt pathway), seem more promising^25^. Also, ATP-competitive mTOR inhibitors are developed that prevent the feedback-mediated Akt activation^26^. Although the treatment with mTOR inhibitors can have beneficial effects, it is often accompanied with severe side effects such as haematological abnormalities, nephrotoxicity, dermatological diseases, impaired wound healing and metabolic disorders^27^, which makes long lasting treatment difficult.

In conclusion, this study shows that rapamycin can have a direct effect on parietal epithelial cells, which *in vivo* can lead to a short-lasting reduction of PEC proliferation and sclerotic lesions. Our findings point out that an elevated mTOR expression in parietal epithelial cells might contribute to PEC activation and the formation of glomerulosclerosis. However, literature and this study show that within the glomerulus mTOR signalling is tightly regulated, resulting in a narrow therapeutic window of rapamycin. When not used in optimal concentrations, as well as in the favourable time window for treatment, mTOR inhibition can have no beneficial or even deleterious effects. In addition, systemic administration of the inhibitor may have non-specific effects, decreasing mTOR inhibition in other cells resulting in for instance prevention of normal kidney repair functions. Whether ATP-competitive mTOR inhibitors or combination treatments of sirolimus and drugs targeting pathways involved in negative feedback loops could decrease sclerosis formation permanently evoking tolerating side effects has to be investigated.

## Material and Methods

### Cell culture

Immortalized polyclonal human parietal epithelial cells (hPECs) were cultured as described in Kietzmann *et al*.^28^. HPECs were differentiated for 2 weeks at 37 °C, 5% (v/v) CO_2_. Conditionally immortalized glomerular endothelial cells (ciGENCs, clone 1D4) were kindly provided by Simon C. Satchell^29^. Details about the culture conditions can be found online in the supplementary materials.

### Animal studies

**Figure 7:**
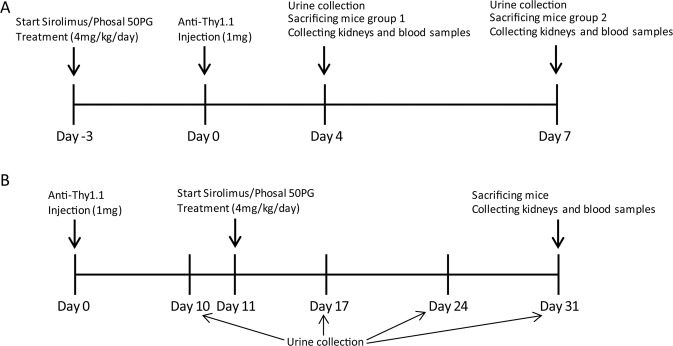
Schematic diagram of the *in vivo* experiments. (**A**) 72 hours prior to anti-Thy1.1 antibody injection animals were daily treated with sirolimus or phosal (vehicle). At day 4, urine was collected from the first group and these mice were sacrificed. Group 2 received treatments for additional 3 days. At day 7, urine was collected from group 2 and these mice were sacrificed. (**B**) Transgenic Thy.1.1 mice were injected with the anti-Thy1.1 antibody and urine was collected after 10 days. From day 11 onwards, mice were treated with sirolimus or the vehicle daily. Additional urine was collected at day 17, day 24 and day 31. Mice were sacrificed at day 31.

All experiments were performed using 6–8 weeks old male Thy1.1 transgenic mice from own breeding. These mice have been backcrossed into the C57BL/6 J for 10 generations. The animal experiments were approved by the Animal Ethics Committee of the Radboud University Nijmegen and performed in accordance with the European Communities Council Directive (86/609/EEC). The setup of the animal experiments is shown in Fig. 7. More details can be found online in the supplementary materials.

### Albumin/Creatinine ratio (ACR)

Urine albumin of the mice was determined using the Mancini immunodiffusion method. The creatinine values were determined at the Radboudumc laboratory of diagnostics (RLD), Radboudumc, Nijmegen, the Netherlands, using a modulation analyser Cobas C8000-1/-2 (Roche Diagnostics).

### Western blotting

RIPA buffer (Cell signaling, #9806) supplemented with 1 mM protease inhibitor phenylmethylsulfonyl fluoride (PMSF) was used for protein extraction of hPECs and ciGENCs. Protein concentrations were determined using the Pierce™ BCA Protein Assay Kit (Thermo Fischer Scientific). Samples were prepared using 4x Laemmli buffer (Bio-Rad) supplemented with beta-mercaptoethanol. Per sample, 25 µg protein was loaded on a 12% (v/v) Sodium Dodecyl Sulphate-Polyacrylamide Gel Electrophoresis (SDS-PAGE) gel and transferred to a nitrocellulose membrane. Antibodies used to detect the pS6RP, the S6RP and the housekeeping gene γ-tubulin are shown in Table 1. These antibodies are well established and commonly used^18,19,21,30–33^. The fluorescent signal was analysed with the Odyssey Imaging System (LI-COR Biosciences) and Odyssey Image studio Software (version 4.0).

**Table 1:** Primary and secondary antibodies used for western blotting and tissue staining.

Primary antibody	Dilution	Secondary antibody	Dilution
P-S6 Ribosomal Protein (S240/244) Rabbit Antibody (Cell Signaling, 2215 S)	1:1,000	Alexa Fluor 680 goat anti-rabbit IgG (H + L) (Life technologies, A21076)	1:10,000
S6 Ribosomal Protein (5G10) Rabbit monoclonal Antibody (Cell Signaling, 2217)	1:5,000	Alexa Fluor 680 goat anti-rabbit IgG (H + L) (Life technologies, A21076)	1:10,000
Anti-γ-tubulin antibody produced in mouse, (Sigma-Aldrich, Clone GTU-88, T6557)	1:10,000	Alexa Fluor 790 donkey anti-mouse IgG (H + L) (Thermo Fischer Scientific, A11371)	1:10,000
Synaptopodin (goat-anti human, sc-21537, Santa Cruz)	1:200	Alexa Fluor donkey anti-goat 546 (11056, ThermoFisher), Alexa Fluor donkey anti-goat IgG 488 (H + L) (A11055 ThermoFisher)	1:200
Desmin (mouse IgG1, DAKO, M076)	1:200	Alexa Fluor goat anti-mouse 488 (IgG1) (A21121, ThermoFisher), 4% mouse serum added to PBS-BSA (1% (w/v))	1:200
P-S6 Ribosomal Protein (S235/236)(Rabbit Ab, #2211 S, Cell signaling)	1:100	HRP conjugated stock solution (goat –anti rabbit HRP conjugated antibody, diluted 30 min incubation, Tyramide antibody (label 647), diluted in amplification buffer, 10 min incubation	HRP: 1:100
			Tyramide: 1:100
SSeCKS (made in rabbit, kindly provided by Prof E. Gelman)	1:500	Alexa Fluor donkey anti-rabbit IgG 568 (H + L) (A10042 ThermoFisher)	1:200
CD34 anti-mouse antibody (CD34, Mouse, mAb MEC14.7, Rat IgG2a, HM1015)	1:100	Alexa Fluor goat anti-rat 647 (H + L) (A21247, Thermofischer)	1:200
Ki-67, Rabbit Monoclonal Antibody, clone sp6, RM-9106-S, Thermo Scientific)	1:100	Alexa Fluor donkey anti-rabbit 488 (H + L) (A21206, Thermofischer)	1:200
DACH1 antibody (HPA012672, Sigma-Aldrich)	1:100	Alexa Fluor donkey anti-rabbit (H + L) (A3157, Thermofischer)	1:200

### Bromodeoxyuridine (BrdU) assay

We performed the BrdU proliferation ELISA, according to manufacturer’s protocol (BrdU Cell Proliferation ELISA Kit, Abcam, ab126556). Cells were treated with different rapamycin concentrations and their respective DMSO controls for 24 hours. The BrdU antibody was incubated for 15 hours. The hPECs were seeded in a density of 20,000 cells/cm^2^ two days prior to rapamycin or DMSO exposure, ciGENCs were seeded in a density of 20,000 cells/cm^2^ 4 days before starting the experiment.

### Tissue staining

All stainings were performed on 4 µm formalin-fixed paraffin-embedded (FFPE) kidney sections. Antigen retrieval was performed in citrate buffer (pH 6) or Tris-EDTA buffer (pH 9) at boiling point for 13 minutes. Details about primary- and matching secondary antibodies used are described in Table 1. Fluorescent stained slices were mounted in DAPI Fluoromount-G® (SouthernBiotech). Co-staining of synaptopodin and desmin was performed to detect injured podocytes. The synaptopodin antibody used was detected with Alexa Fluor donkey anti-goat 546 followed by a 10% (v/v) goat serum in PBS blocking step. Next, the secondary antibody against desmin was applied. To assess pS6RP in podocytes and PECs a triple staining was performed. The P-S6 Ribosomal Protein (S235/236) antibody was visualized using the Tyramide Signal Amplification (TSA) kit #16 (Molecular probes, life technologies, T20926). Subsequently podocytes and PECs were stained with antibodies against synaptopodin and SSeCKS, respectively. To detect proliferating endothelial cells, sections were incubated with CD34 and ki-67 antibodies. Secondary antibodies were used as shown in Table 1. For the detection of podocyte nuclei, sections were incubated with the nuclear marker DACH1 combined with the podocyte maker synaptopodin. The tuft area was measured using CaseViewer software (3DHISTECH Ltd.) and DACH1 positive cells in the glomeruli were counted. A minimum of 48 glomeruli and a maximum of 110 glomeruli per mouse were scored. From each scored glomerulus we calculated the ratio of DACH1 positive podocytes per tuft area (mm^2^) and took the average of these ratios for each mouse.

For detecting ki-67 positive cells immunohistochemically, endogenous peroxidases as well as endogenous biotin, biotin receptors, and avidin were blocked using 3% (v/v) hydrogen peroxide and the Avidin/Biotin blocking Kit (Vector laboratories SP-2001). As primary antibody anti-ki-67 antibody (Table 1) was used, which was detected using a biotinylated Goat Anti-Rabbit IgG Antibody (Vector Laboratories, BA-1000) and the VECTASTAIN® Elite®ABC-HRP Kit (Vector laboratories, PK-6100). The 3,3′-diaminobenzidine (DAB) horseradish peroxidase was used as substrate. Periodic acid–Schiff (PAS) staining without haematoxylin stain was performed afterwards. Per mouse, the ki-67 positive cells were counted in a minimum of 57 and a maximum of 114 glomeruli.

A PAS staining was performed to identify affected glomeruli. A minimum of 58 glomeruli and a maximum of 122 glomeruli per mouse were scored.

### Statistics

The differences between sirolimus and phosal treated mice concerning podocyte count, glomerular proliferation, the percentage of affected glomeruli and desmin expression was statistically tested using a two-tailed Mann-Whitney U test with a confidence interval of 95%. Differences in the albumin/creatinine ratio was tested using a 2-way ANOVA including a Bonferroni post-test for the first experiment and for the second experiment a two-tailed Mann-Whitney U test was used. For the *in vitro* results we used a One-way ANOVA with a Bonferroni’s Multiple Comparison Test. A P value below 0.05 was considered as significant.

## Supplementary information

Supplementary materials.

## Data Availability

The datasets generated during and/or analysed during the current study are available from the corresponding author on reasonable request. Additional information and data is available from the corresponding author on reasonable request.
