# Supplementary material for: Inhibition of mTOR delayed but could not prevent experimental collapsing focal segmental glomerulosclerosis
Source: Sci Rep. 2020 May 22;10:8580. doi: 10.1038/s41598-020-65352-y (PMC7244565; doi:10.1038/s41598-020-65352-y)
Supplement: Supplementary file 1 — Supplementary materials. [file 41598_2020_65352_MOESM1_ESM.docx]

## Inhibition of mTOR delayed but could not prevent experimental collapsing focal segmental glomerulosclerosis

## Supplementary data

Laura Miesen^1^, Jennifer Eymael^1^,  [Shagun Sharma](http://www.abstracts2view.com/asn_2016/view_disclosure.php?usr_id=13967)^[2,3](http://www.abstracts2view.com/asn_2016/view_disclosure.php?usr_id=13967)^[,](http://www.abstracts2view.com/asn_2016/view_disclosure.php?usr_id=13967) [Markus A. Loeven](http://www.abstracts2view.com/asn_2016/view_disclosure.php?usr_id=11822)^[3](http://www.abstracts2view.com/asn_2016/view_disclosure.php?usr_id=11822)^[, Brigith Willemsen](http://www.abstracts2view.com/asn_2016/view_disclosure.php?usr_id=11822)^1^, [Marinka Bakker-van Bebber](http://www.abstracts2view.com/asn_2016/view_disclosure.php?usr_id=13996)^3^, Fieke Mooren^1^, Catherine Meyer-Schwesinger^4^, [Henry Dijkman](http://www.abstracts2view.com/asn_2016/view_disclosure.php?usr_id=11828)^1^, [Jack F.M. Wetzels](http://www.abstracts2view.com/asn_2016/view_disclosure.php?usr_id=385)^3^, Jitske Jansen^1,5^, [Johan van der Vlag](http://www.abstracts2view.com/asn_2016/view_disclosure.php?usr_id=9561)^3,α^ and [Bart Smeets](http://www.abstracts2view.com/asn_2016/view_disclosure.php?usr_id=3778)^1,α,^*

^1^Department of pathology, Radboud Institute for Molecular Life Sciences, Radboud university medical center, Nijmegen, the Netherlands, ^2^School of Biomedical Sciences, University of Plymouth, Plymouth, UK, ^3^Department of Nephrology, Radboud Institute for Molecular Life Sciences, Radboud Institute for Health Sciences, Radboud university medical center, Nijmegen, The Netherlands, ^4^Institute of Cellular and Integrative Physiology, Center for Experimental Medicine, University Medical Center Hamburg -Eppendorf, Hamburg, Germany, ^5^Department of pediatric nephrology, Radboud Institute for Molecular Life Sciences, Radboud university medical center, Amalia Children’s Hospital, Nijmegen, the Netherlands, ^α^Contributed equally

*Corresponding author:

Bart Smeets, PhD

Radboud university medical center

Radboud Institute for Molecular Life Sciences

Department of Pathology

P.O. Box 9101

6500 HB Nijmegen, the Netherlands

Phone: +31 24 3614382

Fax: +31 24 3635125

[Bart.Smeets@radboudumc.nl](mailto:Bart.Smeets@radboudumc.nl)

The provided supplementary data contains the following information: Supplementary material and methods, supplementary figures and legends to supplementary figures.

## Supplementary material and methods

## Setup animal studies

To induce collapsing FSGS in the Thy.1.1 mice, 1mg anti-Thy1.1 monoclonal antibody (mAb, 19XE5) was injected.

Experiment 1: Three days prior to the anti-Thy1.1 mAb injection, daily administration of sirolimus (4mg/kg/day in 0.1ml phosal 50PG,Pfizer Canada) or the vehicle phosal 50PG (0.1ml) via oral gavage was started. Treatment with sirolimus or the vehicle continued until day of sacrifice. The mice were divided into 2 groups, both consisting out of sirolimus and phosal treated mice. At day 0, all mice of group 1 and 2 were injected with a single iv injection of anti-Thy1.1 (1mg). Twenty-four-hour urine collection of the first group of mice was performed at day 3 and these mice were sacrificed at day 4. From the second group, urine was collected at day 6 and the mice were sacrificed at day 7. Before sacrificing the mice, blood collection was performed under anesthesia via orbital puncture. After cervical dislocation, kidneys were removed.

Experiment 2: At day 0, mice were injected with a single i.v. injection of anti-Thy1.1 (1.1mg). Eighteen-hour urine collection was performed at day 10, 17, 24 and 31. At day 10, treatment with sirolimus (4mg/kg/day in 0.1ml phosal 50PG, Pfizer Canada) or phosal 50PG (0.1ml) via oral gavage was started. Treatment with sirolimus or vehicle was continued until the day of sacrifice (day 31). Before sacrificing the mice, blood collection was performed under anesthesia via orbital puncture. After cervical dislocation, kidneys were removed.

## Culture conditions conditionally immortalized glomerular endothelial cells

Conditionally immortalized glomerular endothelial cells (ciGENCs, clone 1D4) were kindly provided by Simon C. Satchell. CiGENCs cultured in fibronectin coated T75 flasks in MCDB 131 basal medium (Life Technologies 10372019) supplemented with 1 µg/ml Ascorbic Acid (Sigma-Aldrich A4544), 0.2 µg/ml Hydrocortisone (Sigma-Aldrich H0888), 4 mM GlutaMAX supplement (Life Technologies 35050061), 30 µg/ml Gentamicin (Sigma-Aldrich G1397), 15 ng/ml Amphotericin B (Sigma-Aldrich A2942), 5 ng/ml recombinant human Epidermal growth factor (EGF) (R&D systems 236-EG), 10 ng/ml recombinant human basic Fibroblast Growth Factor (bFGF) (Merck GF003), 20 ng/ml recombinant human LR3 IGF-I (IGF) (R&D systems 8335-G1), 0.5 ng/ml recombinant human Vascular Endothelial Growth Factor 165 (VEGF) (ThermoFisher RP-8662) and fetal calf serum (FCS, Biochrom A.G.). CiGENCs were cultured under permissive conditions at 33°C, 5% (v/v) CO_2_, and were differentiated at 37°C, 5% (v/v) CO_2_, for 5 days.

## Supplementary figures

###
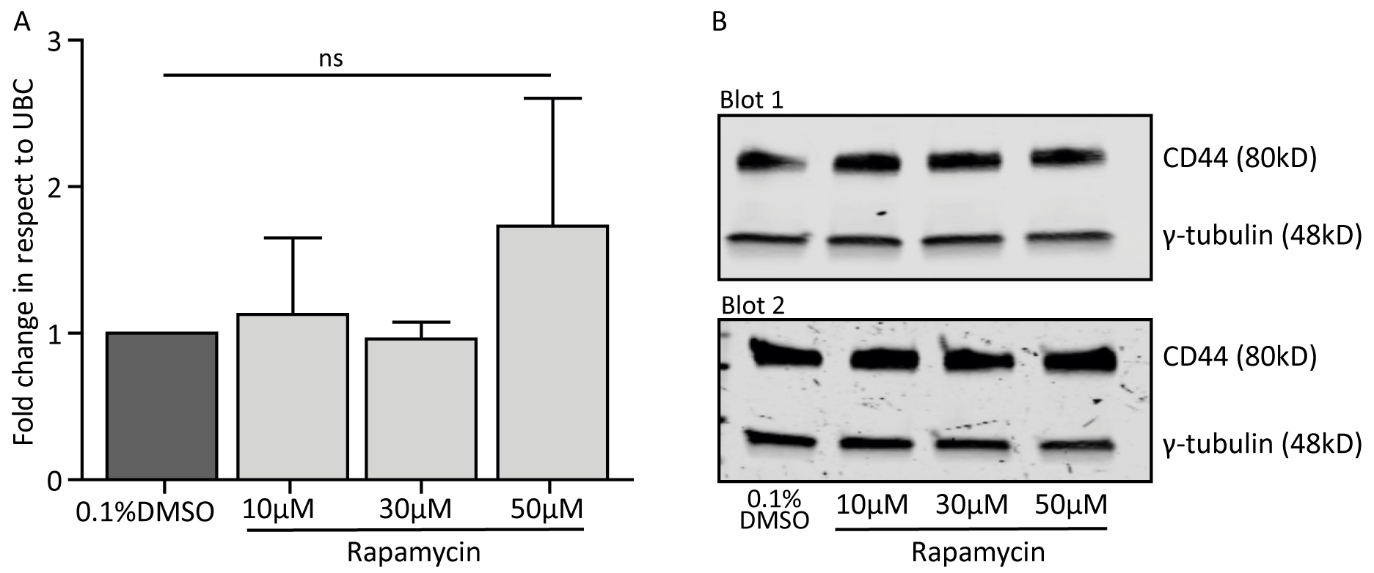
Supplementary figure S1:

##
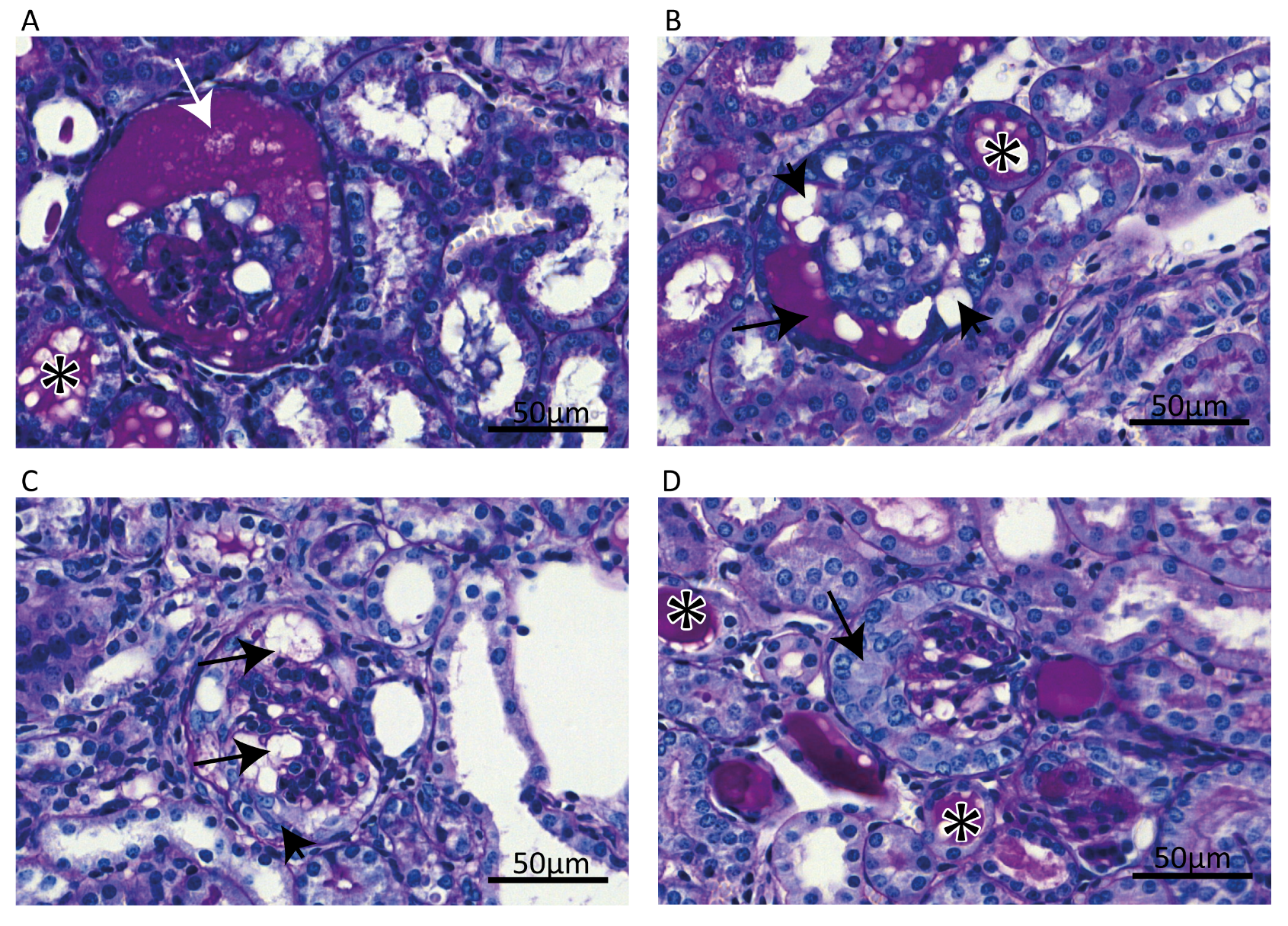
Supplementary figure S2:

##
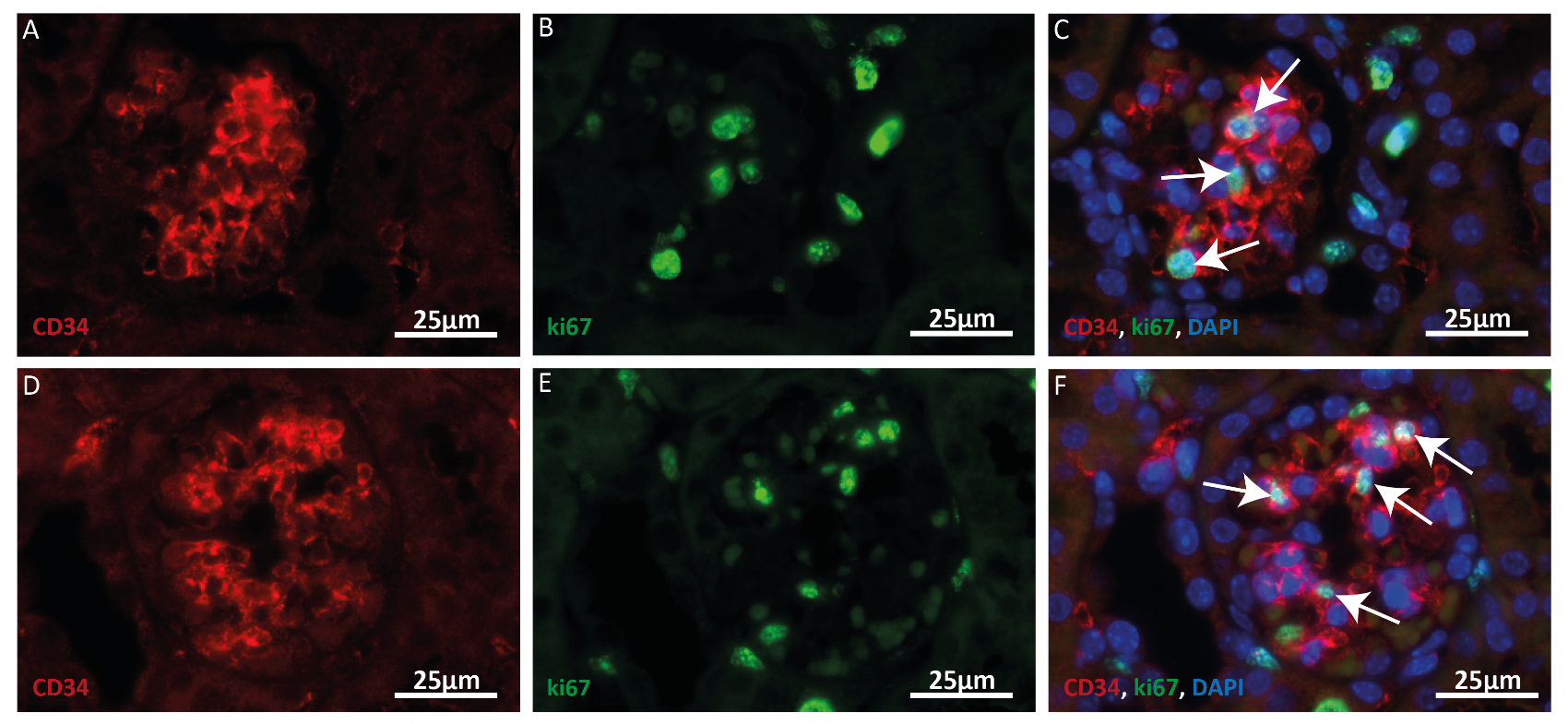
Supplementary figure S3:

### Supplementary figure S4:


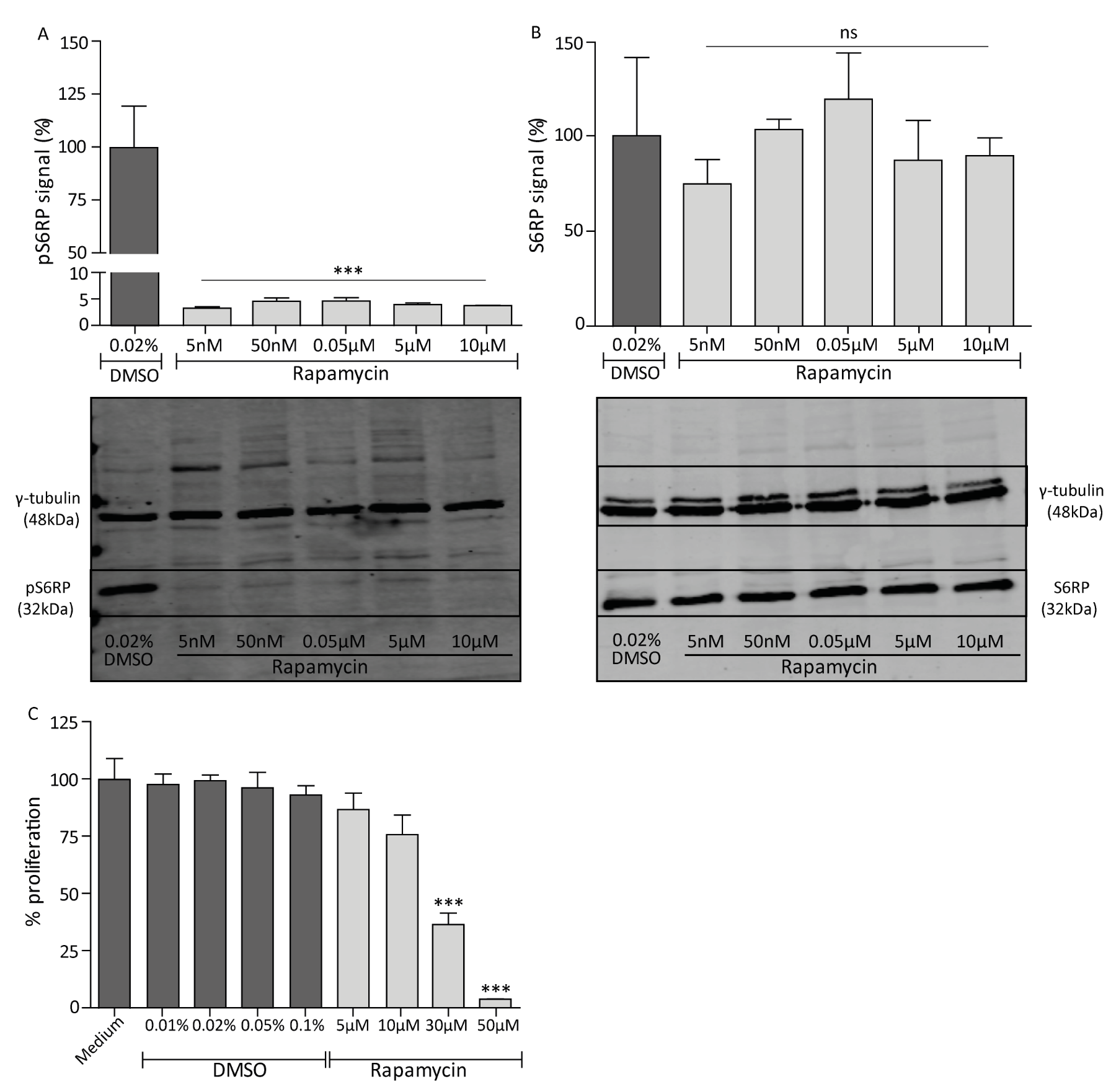


### Full-length western blots:

### Supplementary figure S5:


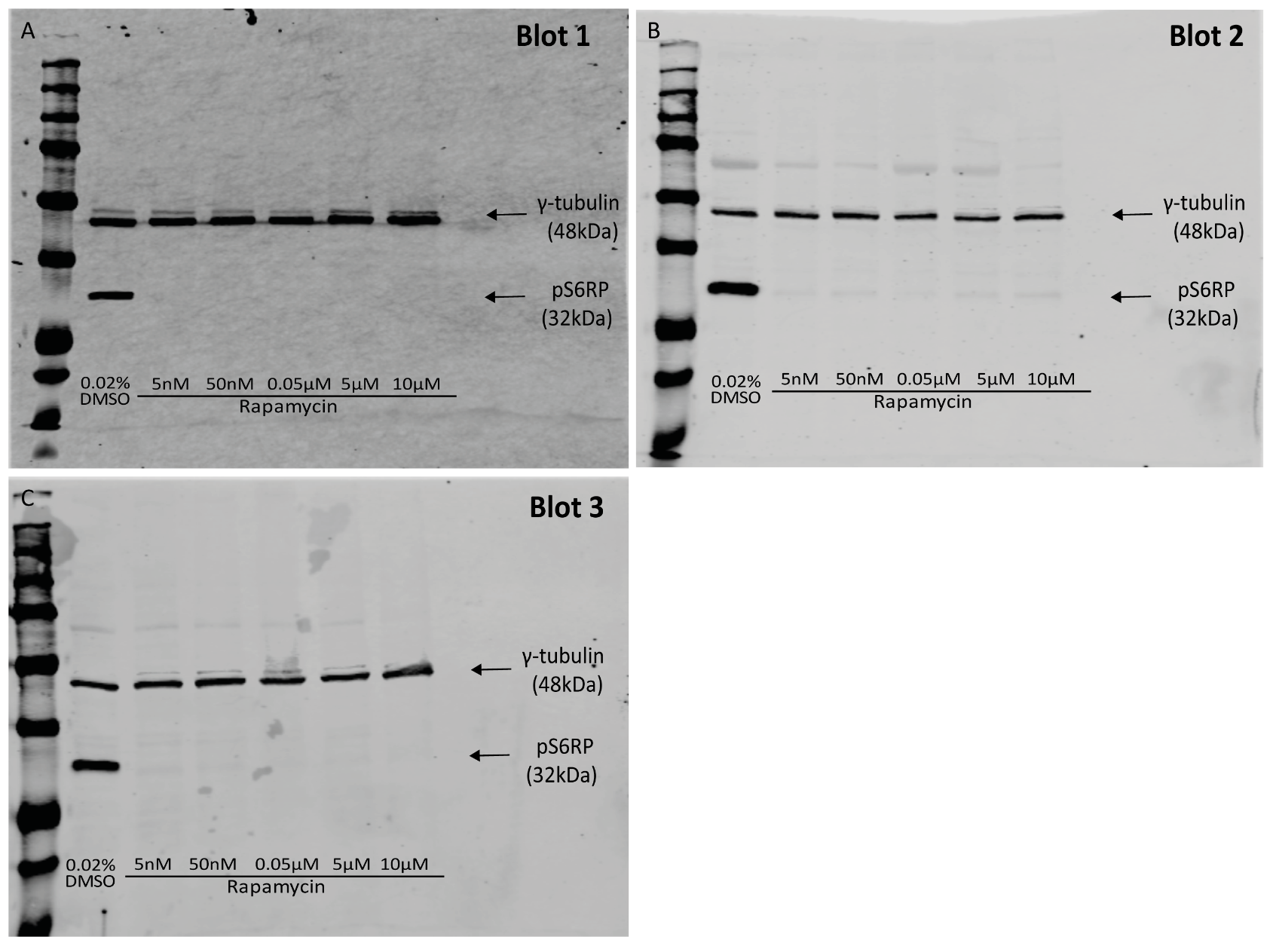


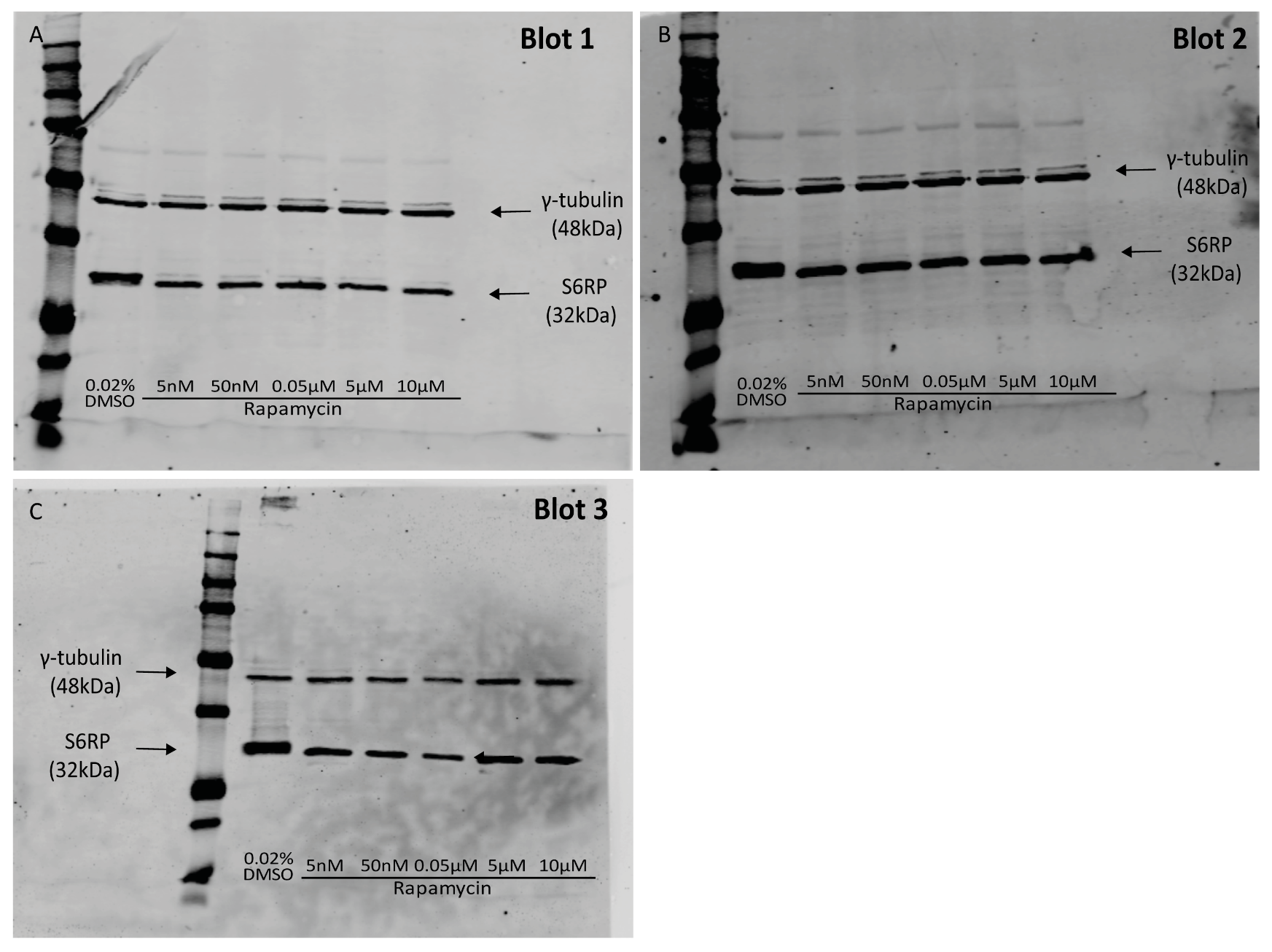
Supplementary figure S6:

##
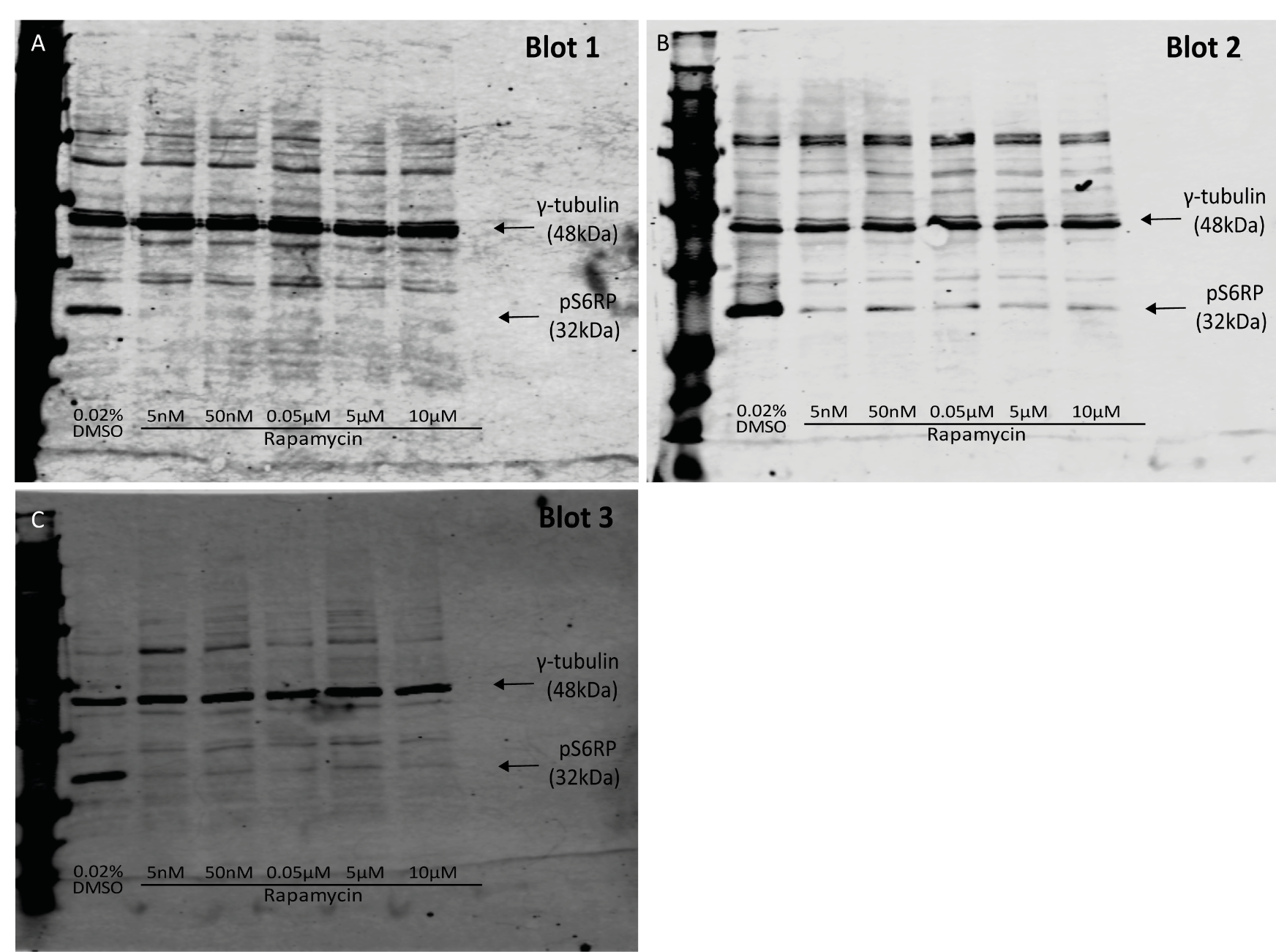
Supplementary figure S7:

## Supplementary figure S8:


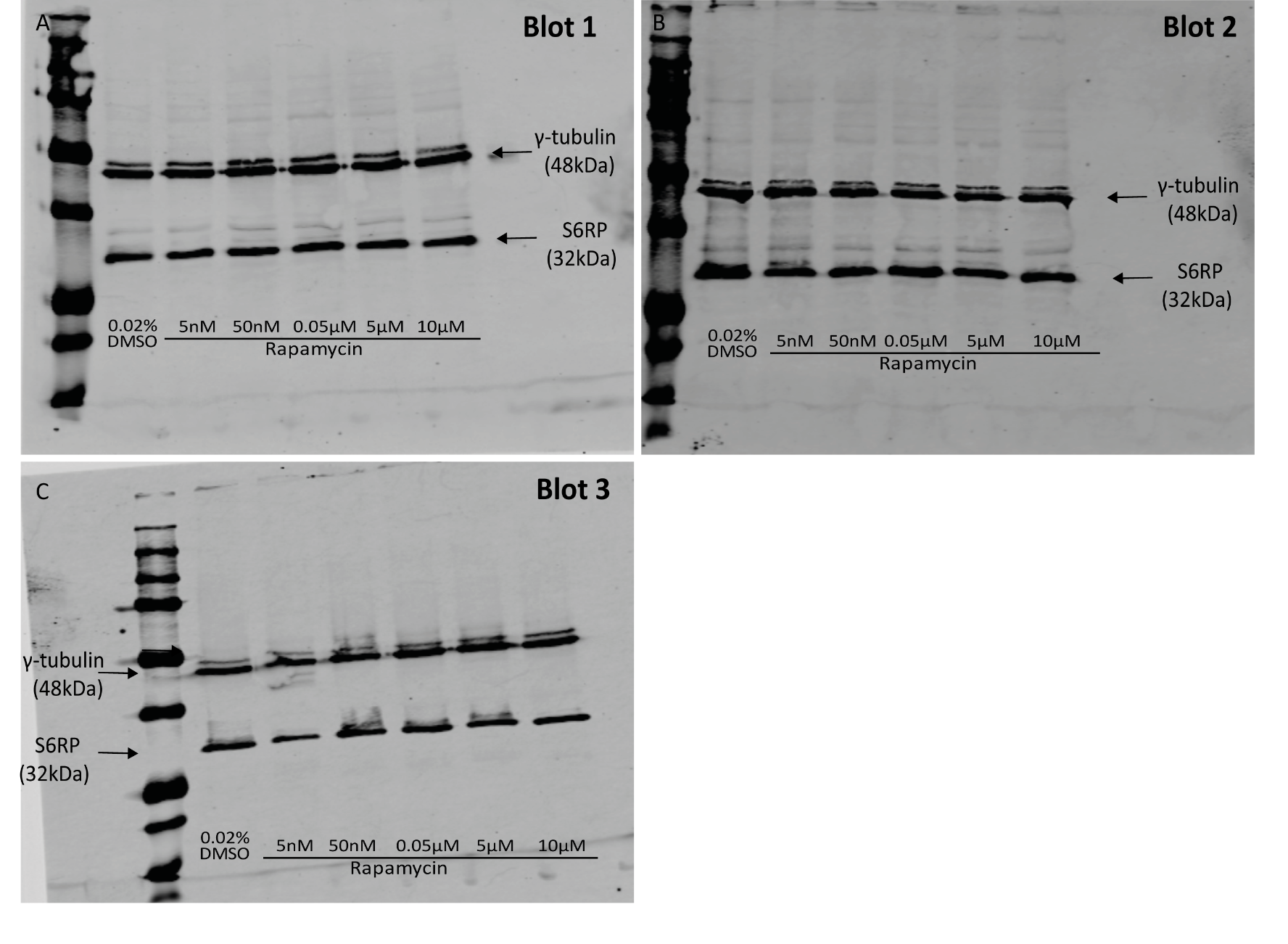


## `

## Legends to Supplementary figures

**Supplementary figure S1| CD44 expression does not change in hPECs treated with rapamycin.** (A) Gene expression of CD44 of hPECs treated with 10µM, 30µM and 50µM did not change significantly. Mean with SEM is shown (n=3), ns P≥0.05, One-way ANOVA, Bonferroni’s multiple comparison test. (B) Protein expression of CD44 stays stable in hPECs treated with rapamycin. CD44 and γ-tubulin signal is shown of 2 blots from 2 independent experiments.

**Supplementary figure S2| Examples of affected glomeruli of anti-Thy.1.1 mice at day 4 after disease induction.** (A) Swollen glomerulus with huge hyalinosis deposition (arrow). Tubuli show protein casts (asterisk). (B) Glomerulus with vacuolization (arrowhead) and hyalinosis (arrow). Tubuli show protein casts (asterisk). (C) Glomerulus with vacuolization (arrow) and cell proliferation (arrowhead). (D) Glomerulus showing PEC proliferation (arrow). Tubuli show protein casts (asterisk).

**Supplementary figure S3| Glomerular endothelial cell proliferation in anti-Thy1.1 mice.** Glomeruli of 2 different mice are presented (A-C and D-F). (A, D) Glomerular endothelial cells were visualized by CD34 staining (red). (B, E) Cell proliferation was reflected by a ki-67 staining (green). (C) Merged images of A and B are shown. Nuclei are visualized with DAPI (blue). The depicted glomerulus shows ki-67 signal in the nuclei of CD34 positive cells (arrows), indicating endothelial cell proliferation. (F) Merged images of D and E are shown. Nuclei are visualized with DAPI (blue). The depicted glomerulus shows ki-67 signal in the nuclei of CD34 positive cells (arrows), indicating endothelial cell proliferation.

**Supplementary figure S4| Rapamycin inhibits mTOR signaling and cell proliferation in ciGENCs**. (A) mTOR signalling is presented as pS6RP expression. Protein expression of pS6RP was significantly reduced in ciGENCs treated with 5nM-10µM rapamycin compared to cells treated with 0.02% (v/v) DMSO (vehicle) for 24 hours. Quantitative analysis was performed using the results of blots of 3 different experiments (n=3). An example of the protein expression of γ-tubulin and pS6RP of one of the three western blots is shown underneath the graph. Protein expression is shown from cells treated with the DMSO control and 5nM-10µM rapamycin. The signal of γ-tubulin and pS6RP shown are cropped and marked with a black box. All full-length blots are presented in Supplementary figure S7. (B) No significant reduction of the non-phosphorylated S6RP was observed in rapamycin treated cells after 24 hours. Quantitative analysis was performed using the results of blots of 3 different experiments (n=3). An example of the protein expression of γ-tubulin and S6RP of one of the three western blots is shown underneath the graph. Protein expression is shown from cells treated with the DMSO control and 5nM-10µM rapamycin. The signal of γ-tubulin and S6RP shown are cropped and marked with a black box. All full-length blots are presented in Supplementary S8. Mean with SEM is shown. ***P≤0.001, ns P≥0.05, One-way ANOVA, Bonferroni’s multiple comparison test**.** (C) CiGENC proliferation was significantly inhibited by rapamycin (30-50µM, 24 hours) compared to respective DMSO controls. (n=3), Mean with SEM is shown. ***P≤0.001, ns P≥0.05, Two-tailed Mann-Whitney U test.

**Supplementary figure S5| Rapamycin inhibits mTOR signalling of human immortalized PECs** - **Full-length western blots pS6RP and γ-tubulin.** (A-C) Full-length western blots of pS6RP and γ-tubulin expression of PECs treated with rapamycin. Protein expression of pS6RP was reduced in PECs treated with 5nM-10µM rapamycin compared to cells treated with 0.02% (v/v) DMSO (vehicle) for 24 hours. Expression of pS6RP and γ-tubulin is marked with an arrow.

**Supplementary figure S6| Rapamycin inhibits mTOR signalling of human immortalized PECs** - **Full-length western blots S6RP and γ-tubulin.** (A-C) Full-length western blots of S6RP and γ-tubulin expression of PECs treated with rapamycin. Protein expression of S6RP was not or slightly reduced in PECs treated with 5nM-10µM rapamycin compared to cells treated with 0.02% (v/v) DMSO (vehicle) for 24 hours. Expression of S6RP and γ-tubulin is marked with an arrow.

**Supplementary figure S7| Rapamycin inhibits mTOR signalling in ciGENCs** - **Full-length western blots pS6RP and γ-tubulin.** (A-C) Full-length western blots of pS6RP and γ-tubulin expression of ciGENCs treated with rapamycin. Protein expression of pS6RP was reduced in ciGENCs treated with 5nM-10µM rapamycin compared to cells treated with 0.02% (v/v) DMSO (vehicle) for 24 hours. Expression of pS6RP and γ-tubulin is marked with an arrow.

**Supplementary figure S8| Rapamycin inhibits mTOR signalling in ciGENCs** - **Full-length western blots S6RP and γ-tubulin.** (A-C) Full-length western blots of S6RP and γ-tubulin expression of ciGENCs treated with rapamycin. Protein expression of S6RP was not or slightly reduced in ciGENCs treated with 5nM-10µM rapamycin compared to cells treated with 0.02% (v/v) DMSO (vehicle) for 24 hours. Expression of S6RP and γ-tubulin is marked with an arrow.
